# Supplementary material for: The variation and evolution of complete human centromeres
Source: bioRxiv. 2023 May 30:2023.05.30.542849. Preprint. [Version 1] doi: 10.1101/2023.05.30.542849 (PMC10312506; doi:10.1101/2023.05.30.542849)
Supplement: Supplement 2 [file media-2.pdf]

SUPPLEMENTARY INFORMATION FOR:

The variation and evolution of complete human centromeres

This PDF file includes:

1. **Supplementary Notes 1 and 2**
2. **References**

SUPPLEMENTARY NOTES

Supplementary Note 1. Analysis of the CHM1 karyotype. We first assessed the karyotype of the CHM1 genome using three orthogonal methods: Giemsa staining, spectral karyotyping, and single-cell sequencing of template DNA strands (Strand-seq; **Extended Data Figs. 2,3**). All three methods indicate that the CHM1 cell population is biclonal, with approximately 71% of cells existing in a diploid or near-diploid state and approximately 29% of cells existing in a tetraploid or near-tetraploid state (**Extended Data Figs. 2a,b**). Both diploid/near-diploid and tetraploid/near-tetraploid cells have multiple chromosomal rearrangements, including a translocation between chromosomes 4q35.1 and 11q24.3 (**Extended Data Figs. 2c,d**), a translocation between chromosomes 16q23.3 to 17q25.3 (**Extended Data Figs. 2e,f**), and a loss of chromosome 17 or the chromosome 17 p-arm in a subset of cells (**Extended Data Fig. 2f**). The translocation between chromosomes 4q35.1/11q24.3 is accompanied by a complete deletion of *STOX2* and *ADAMTS15* and partial deletion of the *ADAMTS8* (**Extended Data Fig. 3c**). *ADAMTS15* is predicted to act as a tumor suppressor gene in breast and colorectal cancer[1,2]. Additionally, the translocation between chromosomes 16q23.3 to 17q25.3 results in a novel gene fusion between *CDH13* and *RPTOR* (**Extended Data Fig. 3d**), which are both associated with cancer[3–6] and may contribute to the observed karyotype of the CHM1 cell line.

Supplementary Note 2. Loss of two distinct chromosomal regions in the CHM1 cell line. Mapping of native PacBio HiFi and ONT long-read sequencing data to the CHM1 centromere assemblies reveals a reduction in coverage on the p-arm-proximal side of the chromosome 17 centromere (**Extended Data Fig. 4**), consistent with the loss of the p-arm in a subset of cells (**Extended Data Fig. 2f**). It also reveals a reduction in coverage over a 631-kbp region in the *D13Z2* α-satellite higher-order repeat (HOR) array on chromosome 13 (**Extended Data Fig. 4**), indicating this region is deleted in a subset of cells.

SUPPLEMENTARY INFORMATION REFERENCES

1. Porter, S. *et al.* ADAMTS8 and ADAMTS15 expression predicts survival in human breast carcinoma. *International Journal of Cancer* **118**, 1241–1247 (2006).
2. Viloria, C. G. *et al.* Genetic Inactivation of ADAMTS15 Metalloprotease in Human Colorectal Cancer. *Cancer Research* **69**, 4926–4934 (2009).
3. Toyooka, K. O. *et al.* Loss of expression and aberrant methylation of the CDH13 (H-cadherin) gene in breast and lung carcinomas. *Cancer Res* **61**, 4556–4560 (2001).
4. Sato, M., Mori, Y., Sakurada, A., Fujimura, S. & Horii, A. The H-cadherin (CDH13) gene is inactivated in human lung cancer. *Hum Genet* **103**, 96–101 (1998).
5. Zhu, Q. *et al.* Hypomethylation of RPTOR in peripheral blood is associated with very early-stage lung cancer. *Clin Chim Acta* **537**, 173–180 (2022).
6. Lu, X. *et al.* Meta-analysis of the association between mTORC1-related genes polymorphisms and cancer risk. *Pathol Res Pract* **229**, 153696 (2022).
